# Efficacy of chemotherapy versus surgery as initial treatment for gastric cancer with positive peritoneal cytology

**DOI:** 10.1186/s12957-023-03085-8

**Published:** 2023-07-12

**Authors:** Bailong Li, Rulin Miao, Fei Shan, Shuangxi Li, Yongning Jia, Kan Xue, Zhemin Li, Xiangji Ying, Fei Pang, Yan Zhang, Jiafu Ji, Ziyu Li

**Affiliations:** Key Laboratory of Carcinogenesis and Translational Research (Ministry of Education/Beijing), Gastrointestinal Cancer Center, Peking University Cancer Hospital & Institute, Haidian District, 52 Fucheng Road, Beijing, 100142 China

**Keywords:** Gastric cancer, Positive peritoneal cytology, Preoperative chemotherapy, Gastrectomy, Prognosis

## Abstract

**Background:**

The prognosis of gastric cancer (GC) patients with positive peritoneal cytology (CY1) without other distant metastasis is poor, and there are no standard treatment strategies. Our study aimed to compare the survival outcomes of CY1 GC patients receiving chemotherapy or surgery as initial treatment.

**Methods:**

From February 2017 to January 2020, clinical and pathological data of patients diagnosed with CY1 GC without other distant metastasis in the Peking University Cancer Hospital was reviewed. Patients were divided into two groups: chemotherapy-initial group and surgery-initial group. In chemotherapy-initial group, patients received preoperative chemotherapy initially. According to the treatment response, the patients were divided into three subgroups: conversion gastrectomy group, palliative gastrectomy group, and further systematic chemotherapy group. In surgery-initial group, patients underwent gastrectomy followed by postoperative chemotherapy.

**Results:**

A total of 96 CY1 GC patients were included with 48 patients in each group. In chemotherapy-initial group, preoperative chemotherapy yielded an objective response rate of 20.8% and disease control rate of 87.5%. Conversion to CY0 after preoperative chemotherapy was obtained in 24 (50%) patients. The median overall survival was 36.1 months in chemotherapy-initial group and 29.7 months in surgery-initial group (*p* = 0.367). The median progression-free survival was 18.1 months in chemotherapy-initial group and 16.1 months in surgery-initial group (*p* = 0.861). The 3-year overall survival rates were 50.0% and 47.9%, respectively. In chemotherapy-initial group, twenty-four patients who converted to CY0 by preoperative chemotherapy and received surgery obtained a significantly better prognosis. The median overall survival was still not reached in these patients.

**Conclusion:**

There was no significant difference in survival outcomes between chemotherapy-initial group and surgery-initial group. CY1 GC patients who converted to CY0 by preoperative chemotherapy and received radical surgery could obtain a favorable long-term prognosis. Further investigation should focus on preoperative chemotherapy to eliminate peritoneal cancer cell.

**Trial registration:**

This study is retrospectively registered.

**Supplementary Information:**

The online version contains supplementary material available at 10.1186/s12957-023-03085-8.

## Introduction

Gastric cancer (GC) is one of the leading types of cancer worldwide, and peritoneum is its most common distant metastatic site [1–3]. Positive peritoneal cytology (CY1) is considered as an early stage of peritoneal metastasis and has been defined as M1 disease in the American Joint Committee TNM staging system [4]. The incidence of CY1 in gastric cancer patients varies from 4 to 41% in previous studies [5, 6].

The standard treatment for CY1 GC patients without any other distant metastasis has not been established. In Japan, gastrectomy with D2 lymphadenectomy followed by S-1 adjuvant chemotherapy is suggested based on the results of CCOG0301 trial. The median overall survival (OS) of CY1 GC patients received D2 gastrectomy, and S-1 adjuvant chemotherapy was 705 days in the phase 2 trial [7, 8]. In the National Comprehensive Cancer Network (NCCN) guidelines, systemic chemotherapy or best supportive care is suggested for these patients, and surgery is not recommended as initial treatment for M1 disease [9]. The median OS of CY1 GC patients treated with chemotherapy initially was 1.7 years in previous study [6]. The patients whose cytology status converted from positive to negative by chemotherapy had better disease-specific survival, but the role of radical surgery after conversion chemotherapy was uncertain [2].

The purpose of our study was to compare the survival outcomes of CY1 GC patients without other distant metastasis receiving chemotherapy or surgery as initial treatment.

## Materials and methods

### Patients

By using the prospectively maintained gastric cancer database at gastrointestinal cancer center of Peking University Cancer Hospital, a retrospective search for clinical and pathological data of CY1 GC patients was conducted between February 2017 and January 2020. Patients with the following criteria were screened: (1) histologically proven adenocarcinoma of the stomach, (2) clinical stage of T2–4 or N + without any evidence of distant metastasis except for CY1 according to the 7th AJCC TNM staging system, (3) no previous anticancer treatment, (4) Eastern Cooperative Oncology Group (ECOG) performance status ≤ 2, and (5) after staging laparoscopy and peritoneal lavage, patients diagnosed with positive cytology and no presence of peritoneal carcinomatosis. Exclusion criteria included the following: (1) clinically diagnosed peritoneal carcinomatosis or other distant metastasis, (2) remnant gastric cancer, recurrent gastric cancer, or multi-primary cancers; and (3) requiring emergency surgery. This study was approved by the Institutional Review Board of Medical Ethics Committee of Peking University Cancer Hospital, and informed consent was obtained from each participant.

### Staging laparoscopy

The procedure of staging laparoscopy and peritoneal cytology examination was described previously [10–12]. Three trocars were used to explore the abdominal cavity. Before any manipulation, 250 mL of warm normal saline was infused into the abdominal cavity. At least 100 mL of lavage fluid was collected for cytology examination. Cytological smears were prepared from centrifuged deposits and were examined by pathologists after Papanicolaou staining. CY1 was considered if there were positive or highly suspicious cancer cells seen in the examination. Then the primary tumor and the abdominal cavity were systematically inspected to exclude gross peritoneal disease or any other distant metastasis.

### Treatment procedure

**Fig. 1:**
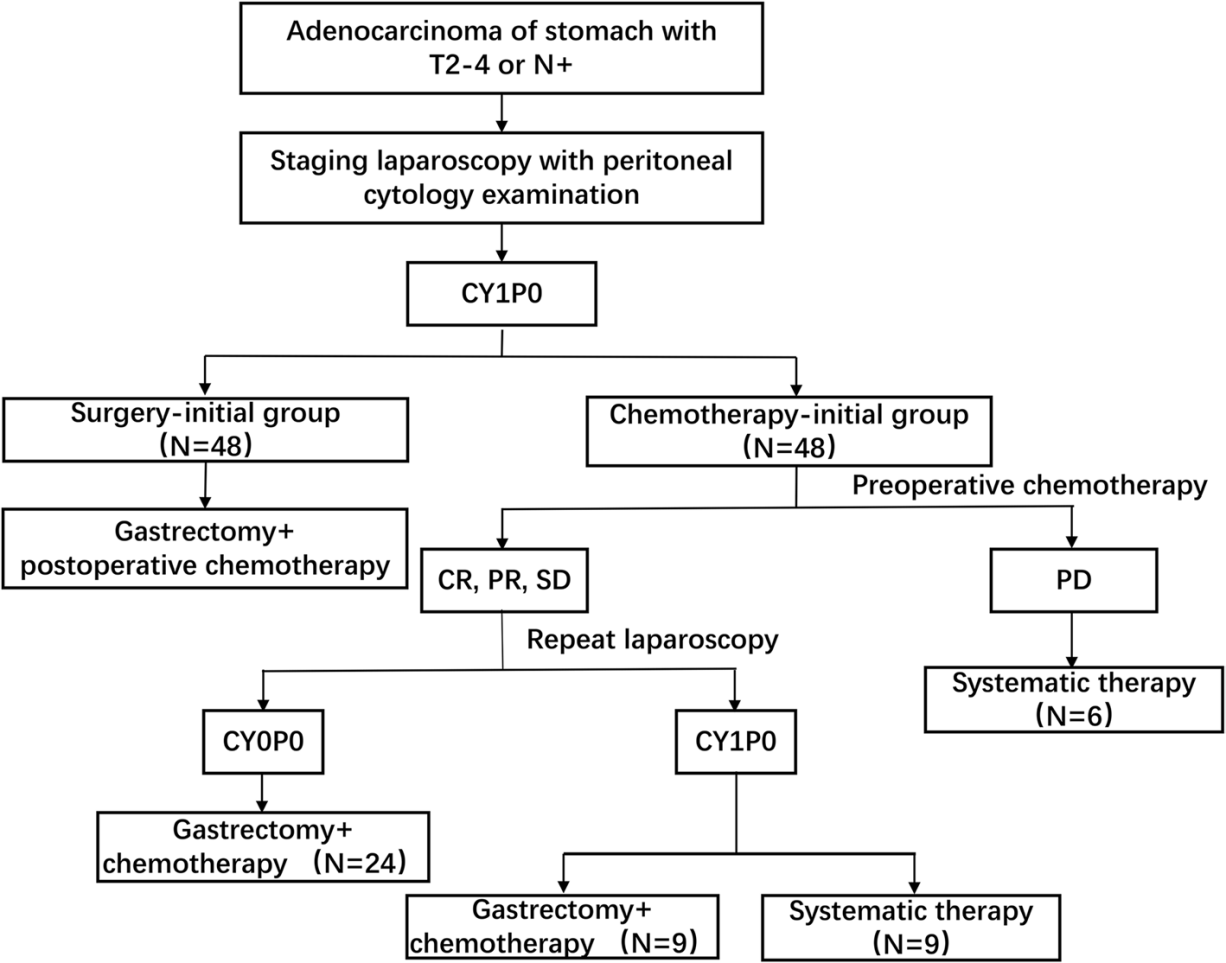
Study flow chart. CR, complete response; PR, partial response; SD, stable disease; PD, progression disease

Two groups were formed based on the initial treatment the patients received: surgery-initial group and chemotherapy-initial group. The choice of treatment strategies was decided through shared decision-making between patients and doctors. The treatment strategies of the two groups were explained to the patients, and the decision was made in advance. During staging laparoscopy, if peritoneal cytology was positive and there was no presence of gross peritoneal disease, the patient would be included in one of the two groups based on previous choice. A flowchart containing the two groups is presented in Fig. 1.

In the surgery-initial group, patients received standard gastrectomy with curative intent according to the Japanese Gastric Cancer Treatment Guidelines [13]. Distal or total gastrectomy with D2 lymph node dissection was performed based on the location of the tumor. The choice of open or laparoscopic surgery was determined by the patient and the surgeon. Combined resection of stomach and other organs was undergone if the primary tumor invaded or adhered to the surrounding visceral organs. After surgery, patients received adjuvant chemotherapy with regimens of fluoropyrimidine and oxaliplatin. S-1/oxaliplatin (SOX) regimen was preferred, and capecitabine/oxaliplatin (CapeOX) regimen and infusional fluorouracil/leucovorin/oxaliplatin (mFOLFOX7) regimen were optional. For SOX or CapeOX regimens, S-1 (40–60 mg orally twice daily for 2 weeks) or capecitabine (1000 mg/m^2^ orally twice daily for 2 weeks) and oxaliplatin (130 mg/m^2^ intravenously on day 1) were recommended every 3 weeks for 8 cycles. For mFOLFOX7 regimen, fluorouracil (2400 mg/m^2^ intravenously for 46 h on days 1–2), leucovorin (400 mg/m^2^ intravenously on day 1), and oxaliplatin (85 mg/m^2^ intravenously on day 1) were recommended every 2 weeks for 12 cycles.

In chemotherapy-initial group, patients received 3–4 cycles of chemotherapy mentioned above as initial treatment. Then tumor response was evaluated according to the response evaluation criteria in solid tumors (RECIST ver. 1.1) by imaging specialists through abdominal contrast-enhanced computed tomography (CT) scan [14]. For patients evaluated as stable disease (SD), partial response (PR), or complete response (CR), a second staging laparoscopy and peritoneal cytology examination were conducted within 3–4 weeks after the last S-1 administration in the last course of chemotherapy. Patients who had converted to negative cytology would receive D2 gastrectomy as described above. After surgery, patients were recommended to receive 4–5 cycles of adjuvant chemotherapy. If the patients were with persistently CY1, newly diagnosed gross peritoneal disease in the repeat laparoscopy, or clinically evaluated progression disease (PD), treatment options, including further systematic chemotherapy or palliative gastrectomy with D2 lymphadenectomy, would be advised based on a multidisciplinary team (MDT) discussion.

In both of the two groups, if the patients had one or more of the following indicators, such as microsatellite instability high (MSI-H), EBER positive, or PD-L1 CPS ≥ 5, chemotherapy plus immunotherapy were strongly recommended. If the patients agreed, chemotherapy plus immunotherapy were employed. If the patients had HER2-positive tumors, chemotherapy was given in combination with intravenous trastuzumab. Trastuzumab was given by intravenous infusion every 3 weeks at a dose of 8 mg/kg on day 1 of the first cycle, followed by 6 mg/kg. In addition, the preoperative treatment responses were evaluated according to the tumor regression grade (TRG) system of NCCN guidelines [9].

### Statistical analysis

Overall survival (OS) was measured from the time of initial staging laparoscopy to death or the latest follow-up date. Progression-free survival (PFS) was calculated from the time of initial staging laparoscopy to the date of the first recurrence in patients who received surgery or to the date that PD was evaluated in chemotherapy-initial group. Postoperative complications were graded according to the Clavien-Dindo classification system [15]. Severe complications were defined as Clavien-Dindo grade 3 or higher. Independent *t*-tests, Mann‒Whitney *U*-tests, chi-square tests, and Fisher’s exact tests were used to determine differences between the two groups’ data. OS and PFS were estimated by the Kaplan–Meier method, and survivals were compared by the log-rank test. Cox proportional hazard models were used to determine predictive factors of survival. Variables with *p*-value < 0.1 in univariate analysis were included in a multivariable model and presented as the hazard ratio (HR) and 95% confidence interval (CI). Variables were expressed in the following manners unless otherwise stated: median (range) or frequency (percentage). All reported *p*-values were two-sided, and *p*-value < 0.05 was considered significant. All statistical analyses were performed in IBM SPSS Statistics, Version 26.0 (IBM Corporation, Armonk, NY, USA).

## Results

### Patient characteristics

A total of 96 patients were identified, including 48 patients in each group. The demographic and clinicopathological characteristics including age, sex, ECOG performance status, BMI, clinical T stage, clinical N stage, histological type, histological grade, Lauren type, and history of abdominal surgery were comparable between two groups. The detailed demographic and clinicopathological characteristics of the two groups are provided in Table 1.

**Table 1:** Patient characteristics in baseline

	Chemotherapy-initial group (*N* = 48)	Surgery-initial group (*N* = 48)	*P*
Mean age (± standard deviation)	58.1 ± 9.8	62.5 ± 10.0	0.055
Sex			0.811
Male	37 (77.1%)	36 (75%)	
Female	11 (22.9%)	12 (25%)	
ECOG PS			0.655
0	35 (72.9%)	33 (68.8%)	
1	13 (27.1%)	15 (31.2%)	
BMI	23 (17–30)	23 (17–30)	0.439
Clinical T stage			0.257
T1–2	1 (2.1%)	3 (6.3%)	
T3	4 (8.3%)	8 (16.7%)	
T4a	32 (66.7%)	27 (56.3%)	
T4b	11 (22.9%)	10 (20.8%)	
Clinical N stage			0.495
N0	3 (6.3%)	7 (14.6%)	
N1	12 (25.0%)	14 (29.2%)	
N2	24 (50.0%)	15 (31.3%)	
N3	9 (18.8%)	12 (25%)	
Histological type			0.315
Adenocarcinoma	28 (58.3%)	35 (72.9%)	
Signet ring-cell carcinoma	18 (37.5%)	12 (25.0%)	
Mucinous adenocarcinoma	2 (4.2%)	1 (2.1%)	
Grade			1.000
G1	2 (4.2%)	2 (4.2%)	
G2	14 (29.2%)	14 (29.2%)	
G3	32 (66.7%)	32 (66.7)	
Lauren type			0.343
Intestinal	18 (37.5%)	19 (39.6%)	
Diffused	18 (37.5)	13 (27.1%)	
Mixed	9 (18.8%)	15 (31.3%)	
NA	3 (6.3%)	1 (2.1%)	
Previous abdominal surgery			0.145
Yes	8 (16.7%)	14 (29.2%)	
No	40 (83.3%)	34 (70.8%)	

*ECOG PS* Eastern Cooperative Oncology Group performance score, *BMI* body mass index

### Efficacy of initial chemotherapy

In the chemotherapy-initial group, 47 patients received SOX regimen, and 1 patient received mFOLFOX7 regimen. Two patients had HER2-positive tumors and received SOX regimen in combination with intravenous trastuzumab. None of the patients received chemotherapy plus immunotherapy preoperatively. With regard to the tumor response, 10 patients (20.8%) were evaluated as PR, 32 patients (66.7%) were evaluated as SD, and 6 patients (12.5%) were PD. The overall response rate was 20.8%. The disease control rate was 87.5%.

Among the patients of PR or SD, peritoneal cytology of 24 patients had converted negative in a second staging laparoscopy and received conversion surgery. Among the remaining 18 patients with persistent CY1 but no gross peritoneal disease, 9 patients received palliative gastrectomy with D2 lymphadenectomy. The remaining 9 patients with persistent CY1 and 6 patients evaluated as PD received further systematic chemotherapy.

### Surgical and pathological outcomes

The surgical and pathological outcomes are summarized in Table 2. In chemotherapy-initial group, a total of 33 patients received surgery. Most patients received open surgery in both groups (83.3% vs. 87.9%, *p* = 0.427). Standard D2 lymphadenectomy was performed in all patients, and the median number of retrieved nodes was 31 and 35 in two groups (*p* = 0.430). Eighteen patients received combined resection, and the details are shown in Supplementary Table 1. R1 resection margins were presented in 2 patients in both groups.

**Table 2:** Surgical and pathological outcomes

	Chemotherapy-initial group (*N* = 33)	Surgery-initial group (*N* = 48)	*P*
Surgical approach			0.427
Open	29 (87.9%)	40 (83.3%)	
Totally laparoscopic	1 (3.0%)	5 (10.4%)	
Laparoscopic-assisted	3 (9.1%)	3 (6.3%)	
Pathological T stage			0.034
T0	1 (3.0%)	0 (0.0%)	
T1	1 (3.0%)	4 (8.3%)	
T2	2 (6.1%)	3 (6.3%)	
T3	20 (60.6%)	15 (31.3%)	
T4a	5 (15.2%)	22 (45.8)	
T4b	4 (12.1%)	4 (8.3%)	
Pathological N stage			0.155
N0	10 (30.3%)	6 (12.5%)	
N1	7 (21.2%)	6 (12.5%)	
N2	5 (15.2%)	15 (31.3%)	
N3a	5 (15.2%)	10 (20.8%)	
N3b	6 (18.2%)	11 (22.9%)	
Neural invasion			0.030
Yes	17 (51.5%)	37 (77.1%)	
No	16 (48.5%)	11 (22.9%)	
Lymphovascular invasion			0.013
Yes	14 (42.4%)	34 (70.8%)	
No	19 (57.6%)	14 (29.2%)	
Combined resection			0.789
Yes	8 (24.2%)	10 (20.8%)	
No	25 (75.8%)	38 (79.2%)	
Number of lymph nodes dissected	31 (12–69)	35 (13–74)	0.430
Postoperative complications			0.938
Yes	8 (24.2%)	12 (25.0%)	
No	25 (75.8%)	36 (75.0%)	
Severe complications			0.574
Yes	4 (12.1%)	4 (8.3%)	
No	29 (87.9%)	44 (91.7%)	

There were no significant differences between the two groups with respect to the incidence rate of postoperative complications (24.2% vs. 25.0%, *p* = 0.938) and severe complications (12.1% vs. 8.3%, *p* = 0.574). The details of postoperative complications are shown in Supplementary Table 2. There was no mortality within 90 days of surgery in both groups.

For pathological outcomes, there were fewer pT4 patients in the chemotherapy-initial group compared to the surgery-initial group (27.3% vs. 54.1%, *p* = 0.034). Pathological N stage was comparable between the two groups (*p* = 0.155). Fewer patients were with lymphovascular invasion (42.4% vs. 70.8%, *p* = 0.013) and neural invasion (51.5% vs. 77.1%, *p* = 0.030) in chemotherapy-initial group. For preoperative treatment response assessment, TRG0, TRG1, TRG2, and TRG3 were achieved in 1, 7, 12, and 13 patients, respectively, in 33 patients who received gastrectomy in chemotherapy-initial group. Pathological outcomes in the chemotherapy-initial group were further assessed based on the CY0P0 and CY1P0 status at the repeat laparoscopy, and the details are shown in Table 3. The pathological outcomes including pathological T stage, pathological N stage, lymphovascular invasion, number of lymph nodes dissected, and TRG were comparable between the patients who converted to CY0P0 and received conversion surgery and patients who were CY1P0 and received palliative surgery. Fewer patients were with neural invasion in the patients who received conversion surgery (35.5% vs. 88.9%, *p* = 0.017).

**Table 3:** Pathological outcomes based on the CY0P0 and CY1P0 status at the repeat laparoscopy

	CY0P0 (*N* = 24)	CY1P0 (*N* = 9)	*P*
Pathological T stage			0.854
T0	1 (4.2%)	0 (0.0%)	
T1	0 (0.0%)	1 (11.1%)	
T2	1 (4.2%)	1 (11.1%)	
T3	16 (66.7%)	4 (44.4%)	
T4a	3 (12.5%)	2 (22.2%)	
T4b	3 (12.5%)	1 (11.1%)	
Pathological N stage			0.950
N0	8 (33.3%)	2 (22.2%)	
N1	5 (20.8%)	2 (22.3%)	
N2	2 (8.3%)	3 (33.3%)	
N3a	4 (16.7%)	1 (11.1%)	
N3b	5 (20.8%)	1 (11.1%)	
Neural invasion			0.017
Yes	9 (37.5%)	8 (88.9%)	
No	15 (62.5%)	1 (11.1%)	
Lymphovascular invasion			1.000
Yes	10 (41.7%)	4 (44.4%)	
No	14 (58.3%)	5 (55.6%)	
Number of lymph nodes dissected	37 (17–74)	30 (13–54)	0.332
TRG			0.714
TRG0	1 (4.2%)	0 (0.0%)	
TRG1	5 (20.8%)	2 (22.2%)	
TRG2	9 (37.5%)	3 (33.3%)	
TRG3	9 (37.5%)	4 (44.4%)	

*TRG* tumor regression grade

### Postoperative treatment

The most commonly used regimens were two-drug regimens based on S-1. The first-line postoperative regimens are shown in Table 4. Two patients in each group had HER2-positive tumors and received postoperative chemotherapy in combination with intravenous trastuzumab. One patient in each group received chemotherapy plus anti-PD-1 antibodies.

**Table 4:** First-line postoperative regimens according to treatment strategies

	Surgery-initial group (*N* = 48)	Conversion surgery group (*N* = 24)	Palliative surgery group (*N* = 9)
S-1	1	-	1
SOX	42	21	7
CapeOX	2	1	-
SOX + H	2	1	1
SOX + anti-PD-1	1	1	-

*SOX* S-1 plus oxaliplatin, *CapeOX* capecitabine plus oxaliplatin, *H* trastuzumab, *anti-PD-1* nivolumab

### Survival outcomes

**Fig. 2:**
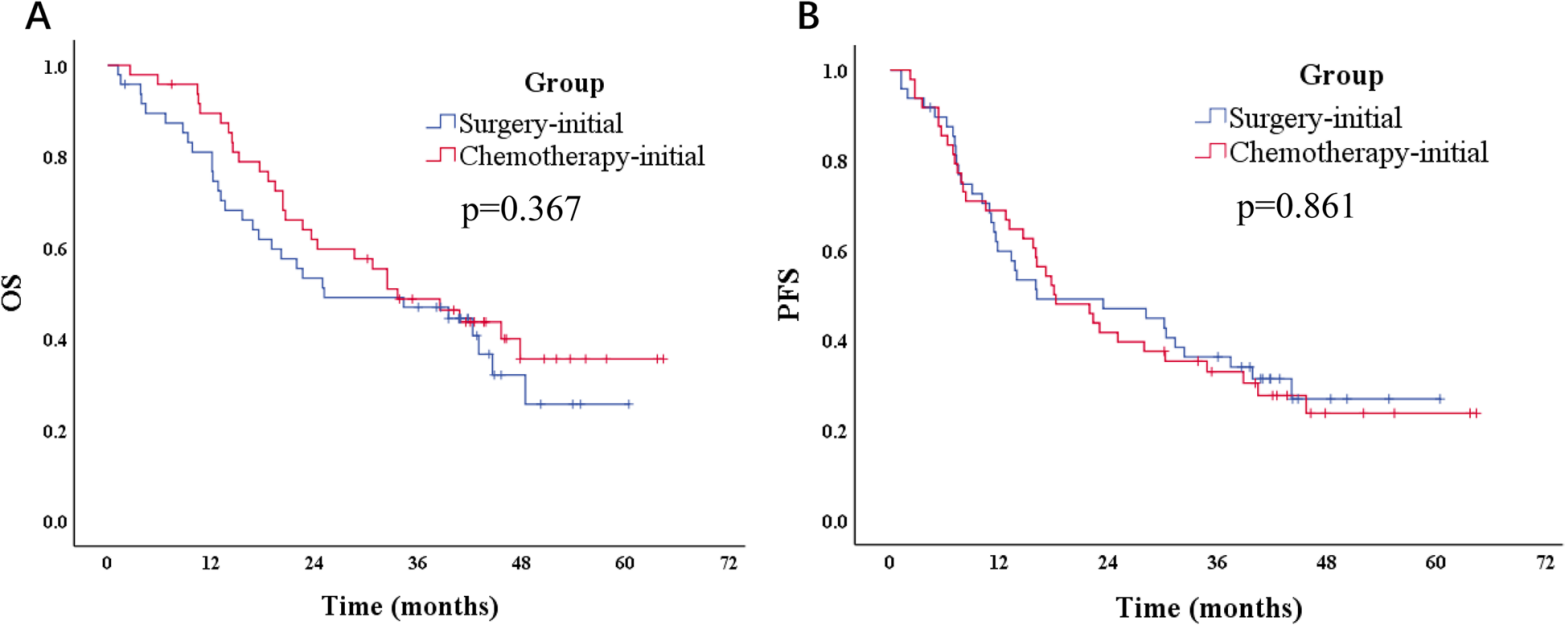
Kaplan–Meier curves for chemotherapy-initial group and surgery-initial group. **A** Overall survival of chemotherapy-initial group and surgery-initial group (log-rank test *p* = 0.367). **B** Progression-free survival of chemotherapy-initial group and surgery-initial group (log-rank test *p* = 0.861)

**Fig. 3:**
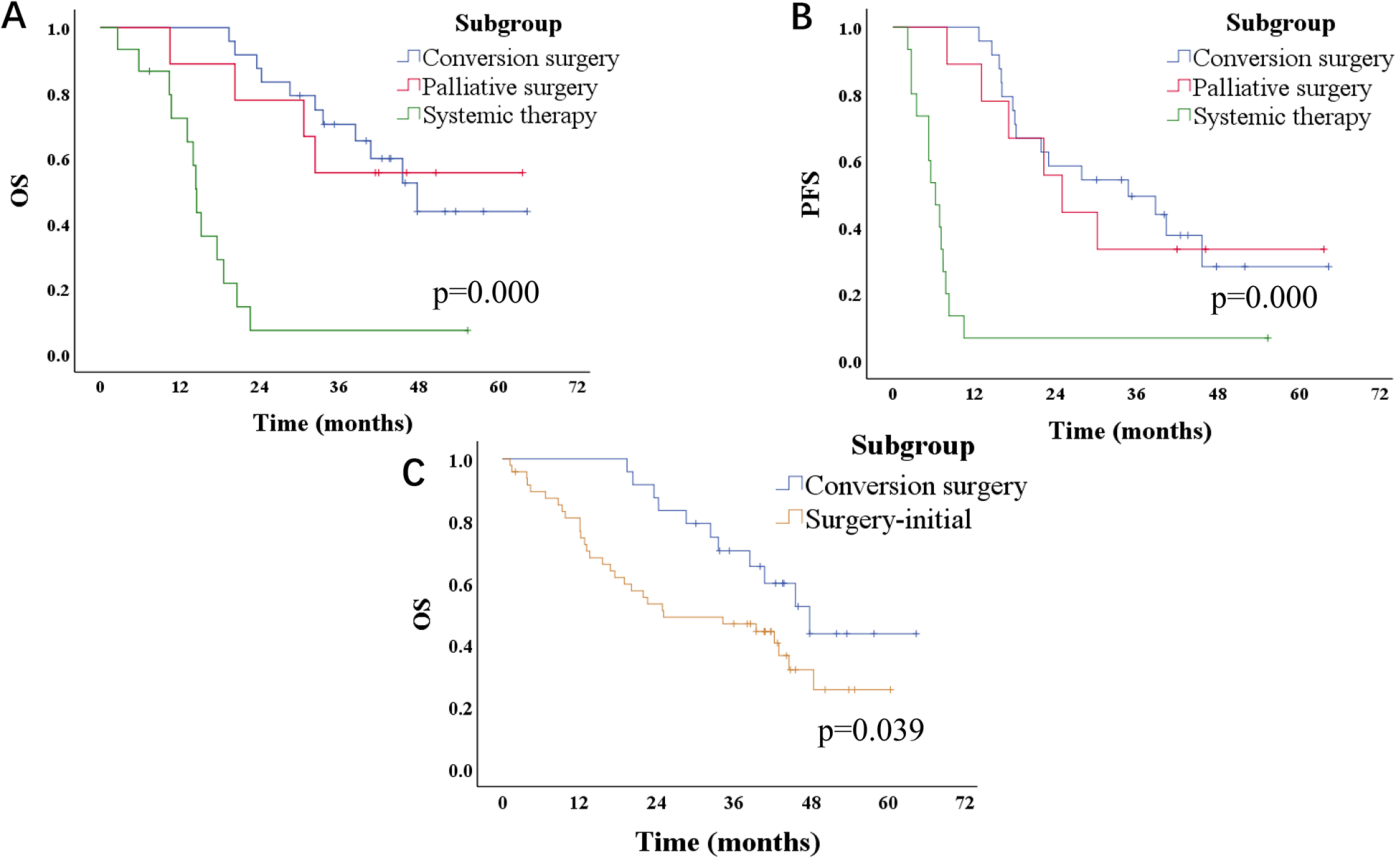
Subgroup analysis. **A** Kaplan–Meier curves for overall survival of conversion surgery group, palliative surgery group, and systematic therapy group (log-rank test *p* = 0.000). **B** Kaplan–Meier curves for progression-free survival of conversion surgery group, palliative surgery group, and systematic therapy group (log-rank test *p* = 0.000). **C** Kaplan–Meier analysis curve for overall survival of patients in surgery-initial group, and patients received conversion surgery (log-rank test *p* = 0.039)

The median follow-up time was 31.6 months. The curves for OS and PFS are presented in Fig. 2. The median OS was 36.1 months for the chemotherapy-initial group and 29.7 months for the surgery-initial group (*p* = 0.367). The median PFS was 18.1 months for the chemotherapy-initial group and 16.1 months for the surgery-initial group (*p* = 0.861). Two patients in surgery-initial group were dead from pneumonia and adverse events after chemotherapy, respectively, without tumor progression. The 3-year OS rates were 50.0% for the chemotherapy-initial group and 47.9% for the surgery-initial group. The 3-year PFS rates were 33.3% and 37.5% in two groups. Among the subgroups of chemotherapy-initial group, the median OS was 14.5 months for patients who received systemic therapy. The median OS was still not reached in patients who converted to CY0 and received conversion surgery and patients who with persistent CY1 and received surgery (*p* = 0.000). The median PFS were 36.8 months, 25.0 months, and 6.3 months for patients who received conversion surgery, patients who received palliative surgery, and patients who received systemic therapy, respectively (*p* = 0.000). Patients who received conversion surgery had a better OS than patients in surgery-initial group (*p* = 0.039, Fig. 3). Among all patients with progression or distant metastasis during follow-up, peritoneum was the most common site (*n* = 45), followed by the liver (*n* = 5), lung (*n* = 5), retroperitoneal lymph nodes (*n* = 5), ovarian (*n* = 3), stomach anastomosis (*n* = 3), spine (*n* = 2), rectum (*n* = 1), urinary bladder (*n* = 1), biliary duct (*n* = 1), adrenal gland (*n* = 1), and small intestine (*n* = 1).

Univariable and multivariable analyses were performed to identify prognostic factors of OS for CY1 GC patients. The details are displayed in Table 5. In all patients, age (*HR* = 1.03, 95% *CI* 0.99–1.06, *p* = 0.090), clinical T4 stage (*HR* = 2.25, 95% *CI* 0.97–5.26, *p* = 0.060), clinical N2–3 (*HR* = 1.84, 95% *CI* 1.05–3.23, *p* = 0.032), signet ring-cell carcinoma histology type (*HR* = 1.94, 95% *CI* 1.14–3.30, *p* = 0.014), G3 grade (*HR* = 2.42, 95% *CI* 1.28–4.47, *p* = 0.007), lymphovascular invasion (*HR* = 3.39, 95% *CI* 1.71–6.71, *p* = 0.000), and neural invasion (*HR* = 3.50, 95% *CI* 1.62–7.56, *p* = 0.000) were associated with worse OS. BMI (*HR* = 0.88, 95% *CI* 0.80–0.96, *p* = 0.003) was associated with better OS. When all these variables were included in an adjusted multivariable model, neural invasion (*HR* = 2.90, 95% *CI* 1.20–6.98, *p* = 0.018) was identified as the only independent prognostic factor of OS.

**Table 5:** Univariate and multivariate analyses for OS with Cox proportional hazard models

Variables	Univariate analysis		Multivariate analysis	
	HR (95% *CI*)	*P*	HR (95% *CI*)	*P*
Age	1.03 (0.99–1.06)	0.090	1.04 (0.99–1.08)	0.060
Sex				
Male	Reference			
Female	1.36 (0.76–2.43)	0.297		
ECOG PS				
0	Reference			
1	1.43 (0.85–2.42)	0.179		
BMI	0.88 (0.80–0.96)	0.003	0.93 (0.83–1.04)	0.179
Treatment decision				
Surgery initial	Reference			
Chemotherapy initial	0.79 (0.47–1.33)	0.382		
Clinical T stage				
T1–3	Reference			
T4	2.25 (0.97–5.26)	0.060	2.92 (0.99–8.62)	0.052
Clinical N stage				
N0–1	Reference			
N2–3	1.84 (1.05–3.23)	0.032	1.14 (0.55–2.36)	0.735
Histologic type				
Adenocarcinoma	Reference			
Signet ring-cell carcinoma	1.94 (1.14–3.30)	0.014	1.73 (0.86–3.46)	0.123
Grade				
G1–2	Reference		Reference	
G3	2.42 (1.28–4.47)	0.007	2.11 (0.91–4.88)	0.081
Lymphovascular invasion				
No	Reference		Reference	
Yes	3.39 (1.71–6.71)	0.000	1.69 (0.79–3.62)	0.177
Neural invasion				
No	Reference		Reference	
Yes	3.50 (1.62–7.56)	0.000	2.90 (1.20–6.98)	0.018

## Discussion

Peritoneal cytology is an independent predictor of survival in patients with gastric cancer [16, 17]. However, there is no high-level evidence from large-scale RCTs to establish a standard treatment for CY1 GC patients. Treatment recommendations are different between guidelines from Western and Eastern countries. Western studies proved that chemotherapy was effective for CY1 GC patients, and surgery is not recommended as initial treatment for M1 disease in NCCN guidelines. Badgwell et al. reported 39 patients diagnosed with CY1 GC at baseline [5]. Twenty-four patients received chemotherapy, while the other 15 patients received palliative therapy only. They reported a significantly improved survival outcome of median OS (16.2 months vs. 7.2 months) and 3-year survival rates (12% vs. 0%) for patients who received chemotherapy. Three patients treated with chemotherapy initially followed by gastrectomy obtained a longer survival. Lorenzen et al. reported 19 patients with CY1 GC and were treated with chemotherapy of cisplatin plus fluorouracil [18]. At a repeat laparoscopy, the cytology status of 7 patients converted negative and exhibited an improved prognosis, with 36.1 months of median OS and 71.4% of 2-year survival rates compared to 9.2 months and 25% in patients with persistently positive cytology. Instead, gastrectomy with lymph node dissection followed by S-1 monotherapy is recommended in guidelines from Japan [7, 19]. The median OS was 705 days, and 5-year OS rates were 26% in CCOG0301 trial. Studies from Japan recommended that gastrectomy followed by S-1 chemotherapy would bring survival benefit to these patients, with 5-year overall survival rates ranged from 18 to 26% and median overall survival from 22.3 to 23.5 months [8, 20, 21]. However, there were few studies to compare the survival outcomes of CY1 GC patients receiving chemotherapy or surgery as initial treatment, and most of the previous studies were with small sample sizes and included patients with limited peritoneal metastasis. Thus, it is necessary to conduct the study to compare the survival outcome of two treatment strategies.

In the present study, both of the groups achieved satisfactory long-term survival, with 36.1 months and 29.7 months of median OS in chemotherapy-initial and surgery initial groups, respectively. Three-year OS rates were 50.0% in the chemotherapy-initial group and 47.9% in the surgery-initial group. These results suggested that GC patients diagnosed with positive peritoneal cytology without other distant metastasis could obtain a decent prognosis through multimodality treatment. Furthermore, it seems reasonable to consider CY1 GC as a target for cure. Nevertheless, there was no statistically significant difference between two groups in OS and PFS in our study. This result was consistent with part of previous studies [22, 23]. In a multi-institutional retrospective study from Japan, the survival outcomes were comparable in initial-Cx and initial-Sx groups for GC patients with CY1 or/and P1a, with median OS of 24.8 months and 24.0 months, respectively [24]. In addition, the subgroup analysis of JCOG0501 trial also did not reveal that preoperative chemotherapy provided meaningful improvement in survival for type 4 or large type 3 gastric cancer patients with CY1 and no other distant metastasis [25].

Of note, our study showed that the patients who converted to CY0 through preoperative chemotherapy and received standard surgery in chemotherapy-initial group obtained a significantly more favorable long-term prognosis than the patients in surgery-initial group. Similarly, a multicenter study of Italian Peritoneal Surface Malignancies Oncoteam conducted on peritoneal metastases patients showed significant survival difference between patients with positive peritoneal cytology received cytoreductive surgery (CRS) plus hyperthermic intraperitoneal chemotherapy (HIPEC) and patients without free peritoneal metastatic cells (10.3 months vs. 44.3 months) [26]. These results suggested that elimination of peritoneal cytology should be the therapeutic goal for these patients. In addition, the prognosis of the two groups in present study was better than that of previously reported [24], and this consequence suggests that chemotherapy of SOX regimen may be a potentially valid regimen using for perioperative treatment for CY1 patients. Preoperative chemotherapy with S-1 plus oxaliplatin yielded an objective response rate of 20.8% and disease control rate of 87.5%. It was reported that 38% patients with CY1 or P1a converted to CY0 after initial chemotherapy, and 51.4% patients treated with docetaxel plus cisplatin plus S-1 (DCS regimen) converted to CY0 which was the highest among all the chemotherapy regimens [24]. In the present study, conversion to CY0 after preoperative chemotherapy was obtained in 50% patients.

Our results showed that the pathological T stage was earlier, and there were less lymphovascular invasion and neural invasion in the chemotherapy-initial group. The downstaging effects of chemotherapy may contribute to abovementioned pathological results. Moreover, in the multivariable analysis, neural invasion was identified as the only independent prognostic factor of OS. We considered that chemotherapy as the initial treatment might be recommended, because it might change the neural invasion to negative, though there was no statistically significant difference in OS between the two groups in the present study. Therefore, more effective preoperative chemotherapy regimens are warranted. In addition, the incidence rates of postoperative complication were similar between chemotherapy-initial group and surgery-initial group, and it proved that surgery was safe for CY1 GC patients treated by preoperative chemotherapy.

In the present study, the treatment options for patients with persistent positive cytology at the repeat laparoscopy included systematic therapy and palliative gastrectomy. According to the results of the CCOG0301 trail, gastrectomy with lymph node dissection leaving no visible disease followed by S-1 chemotherapy could prolong the survival for the patients with CY1 [8]. It was the rationale that part of patients with persistent CY1 received palliative gastrectomy. Notably, in subgroup analysis of chemotherapy-initial group, OS and PFS did not differ significantly between patients who converted to CY0 and received radical surgery and patients who with persistent CY1 after chemotherapy and received palliative surgery. This result was not consistent with the previous studies reported by Lorenzen and Yago [18, 22]. However, it should be noted that the patients who received palliative surgery in abovementioned studies did not receive adjuvant chemotherapy as routine. The patients who received palliative surgery completed postoperative chemotherapy in the present study. In addition, in a previous study, Nakamura et al. proposed that more than five courses of neoadjuvant chemotherapy might be essential to get negative conversion in peritoneal cytology [27]. But in our center, 3–4 courses of preoperative chemotherapy were administered routinely. Therefore, we hold the hypothesis that the possible reason for the comparable survival outcomes in the two subgroups was that patients who received surgery with persistent CY1 converted to CY0 through postoperative chemotherapy, but we could not examine the cytology status of these patients in outpatient. Moreover, the pathological outcomes were also comparable in the two subgroups, including pathological T stage, pathological N stage, lymphovascular invasion, number of lymph nodes dissected, and TRG.

In the clinical practice, clinicians require to be faced with selection of the treatment strategy for CY1 GC patients. According to the survival outcomes in the two groups in present study, it seems that surgery-initial and chemotherapy-initial treatment are both reasonable. Particularly, patients who converted to CY0 are expected to obtain a better prognosis. However, a small proportion of patients in the chemotherapy-initial group may undergo progression during preoperative chemotherapy and have impaired median survival. It is difficult to predict the response to chemotherapy before the initial treatment. Thus, the advances in the more intensive regimens are needed, including immunotherapy and intraperitoneal chemotherapy. Additionally, to our best knowledge, there were few studies conducted investigating the role of circulating metastatic cells in patients with peritoneal metastases from GC. The potential reason for the survival benefit of patients whose cytology converted to negative might be related to the lower malignant tumor biological characteristics. Therefore, it is important to conduct studies exploring these issues and select suitable patients with peritoneal metastases for CRS and HIPEC. Another issue should be established is the optimal number of preoperative chemotherapy courses.

There are several limitations in our study. First, this is a retrospective study, and the selection bias is inevitable. Fortunately, the baseline data of the two groups were comparable. Second, the sample size in the present study is also limited. Third, the present study is a single-center study conducted in Eastern Asian country. Considering the difference of perioperative chemotherapy regimens between Western and Eastern countries, the generalizability of our findings is limited in the centers that S-1 is not applied routinely. Fourthly, all the patients who converted to CY0 in the chemotherapy-initial group received radical surgery in the present study. Whether this part of patients could achieve a long-term survival without surgery is unknown. Despite these limitations, our study provides survival data of these CY1 GC patients receiving chemotherapy or surgery as initial treatment and can contribute to future study design.

## Conclusion

Multimodality treatment is reasonable for the CY1 GC patients without any other distant metastasis. Patients who convert to CY0 by preoperative chemotherapy and receive radical surgery can obtain a favorable long-term prognosis. Further research in medications is urgently needed and should focus on clearing the peritoneal cancer cell.

## Supplementary Information

**Additional file 1: Supplementary Table 1.** Multiple organs resection. **Supplementary Table 2.** Postoperative complications.

## Data Availability

The datasets used and/or analyzed during the current study are available from the corresponding author on reasonable request.

## Abbreviations

GC: Gastric cancer
CY1: Positive peritoneal cytology
OS: Overall survival
PFS: Progression-free survival
CT: Computed tomography
SD: Stable disease
PR: Partial response
CR: Complete response
PD: Progression disease
RECIST: Response evaluation criteria in solid tumors
MDT: Multidisciplinary team
TRG: Tumor regression grade
CRS: Cytoreductive surgery
HIPEC: Hyperthermic intraperitoneal chemotherapy
