# Supplementary material for: Efficacy of chemotherapy versus surgery as initial treatment for gastric cancer with positive peritoneal cytology
Source: World J Surg Oncol. 2023 Jul 12;21:204. doi: 10.1186/s12957-023-03085-8 (PMC10337093; doi:10.1186/s12957-023-03085-8)
Supplement: Supplementary file 1 — Additional file 1: Supplementary Table 1. Multiple organs resection. Supplementary Table 2. Postoperative complications. [file 12957_2023_3085_MOESM1_ESM.docx]

**Supplementary table 1** Multiple organs resection.

|  | Chemotherapy-initial group  (N=8) | Surgery-initial group  (N=10) |
| --- | --- | --- |
| Spleen | 3 | 2 |
| Partial pancreas | - | 1 |
| Partial liver | 1 | - |
| Transverse colon | - | 1 |
| Transverse mesocolon | - | 2 |
| Gall bladder | - | 3 |
| Ovary | 1 | - |
| partial pancreas and spleen | 3 | - |
| Partial pancreas and transverse mesocolon | - | 1 |

**Supplementary table 2:** Postoperative complications.

|  | Chemotherapy-initial group | | Surgery-initial group | | P |
| --- | --- | --- | --- | --- | --- |
| Overall complications |  |  | | 0.938 | |
| Pancreatic leakage | 1 | 3 | |  | |
| Chylous leakage | - | 3 | |  | |
| Gastroparesis | - | 2 | |  | |
| Ileus | - | 1 | |  | |
| Anastomotic leakage | 2 | - | |  | |
| Anastomotic stenosis | 1 | - | |  | |
| Anastomotic bleeding | 1 | - | |  | |
| Abdominal infection | 1 | 1 | |  | |
| Abdominal hemorrhage | - | 1 | |  | |
| Respiratory failure | - | 1 | |  | |
| Pneumonia | 1 | - | |  | |
| Pleural effusion | 1 | - | |  | |
| Severe complications |  |  | | 0.574 | |
| Pancreatic leakage | - | 1 | |  | |
| Abdominal infection | 1 | 1 | |  | |
| Abdominal hemorrhage | - | 1 | |  | |
| Anastomotic bleeding | 1 | - | |  | |
| Anastomotic leakage | 1 | - | |  | |
| Respiratory failure | - | 1 | |  | |
| Pleural effusion | 1 | - | |  | |
